# Downregulation of *WNT11* is associated with bladder tissue fibrosis in patients with interstitial cystitis/bladder pain syndrome without Hunner lesion

**DOI:** 10.1038/s41598-018-28093-7

**Published:** 2018-06-28

**Authors:** Daeheon Choi, Ju-Young Han, Jung Hyun Shin, Chae-Min Ryu, Hwan Yeul Yu, Aram Kim, Seungun Lee, Jisun Lim, Dong-Myung Shin, Myung-Soo Choo

**Affiliations:** 1 Department of Urology, University of Ulsan College of Medicine, Seoul, Korea; 2 Department of Biomedical Sciences, University of Ulsan College of Medicine, Seoul, Korea; 3 Department of Physiology, Asan Medical Center, AMIST, University of Ulsan College of Medicine, Seoul, Korea; 4 Department of Urology, Konkuk University Hospital, Konkuk University School of Medicine, Seoul, Korea

## Abstract

This study assessed the functional role of WNT genes and the association between WNT signalling cascades and fibrosis in interstitial cystitis/bladder pain syndrome (IC/BPS) patients. Twenty-five patients (3 males, 22 females; mean age 59.7 ± 10.9 years), included 7 non-Hunner-type IC (NHIC), 18 Hunner-type IC (HIC), and 5 non-IC (control) groups. The expression of sonic hedgehog, WNT gene family, and genes previously reported as biomarkers for IC/BPS were examined using RT-PCR in biopsy specimens from the mucosa and submucosa layer of the bladder. *WNT2B, WNT5A*, *WNT10A*, and *WNT11* functions in the urothelium were evaluated by silencing in an HBlEpC cell line. Pelvic Pain and Urgency/Frequency Patient Symptom Scale scores, O’Leary-Sant Symptom and Problem Index scores, and Visual Analogue Scores did not differ between the NHIC and HIC groups. However, HIC patients had significantly shorter symptom duration (30.9 vs 70.8 months, p = 0.046), higher daily urinary frequency (16.1 versus 8.5 times, p = 0.006), and smaller bladder capacity (208.6 versus 361.4 ml, p = 0.006) than NHIC patients. Overall WNT gene expression was lower in NHIC than HIC patients. Bladder epithelial tissues from HIC patients were characterised by the downregulation of *WNT11*. Silencing of *WNT11*, *WNT2B*, *WNT5A*, and *WNT10A* in HBlEpCs resulted in fibrotic changes, indicated by fibrotic morphology, increased fibrosis-related gene expression, and nuclear localisation of phosphorylated SMAD2, and increased vimentin and fibronectin levels. Downregulation of *WNT11* results in fibrotic changes of bladder epithelial cells and is associated with the pathogenesis and differential diagnosis of NHIC. Decreased expression of *WNT11* is a potential biomarker for predicting NHIC.

## Introduction

Interstitial cystitis/bladder pain syndrome (IC/BPS) is a distressing, chronic bladder disorder characterised by urinary frequency, urgency, nocturia, and pelvic pain without bacterial infection or an identifiable pathologic cause^1^. The quality of life of IC/BPS patients is often poor. Only a few treatment options have been identified, and there is no cure.

Although investigated in many studies, the pathogenesis of IC/BPS still remains unclear. Previous studies have described the reduction in the glycosaminoglycan (GAG) layer in the bladder of IC/BPS patients^2,3^. An impaired GAG layer can result in an imbalance in urine storage, causing frequent voiding, reduced bladder capacity, and pelvic pain. The pathophysiology of IC/BPS also includes mast cell increases in the bladder^4^. A damaged urothelium can result in activation of mast cells, thus inducing inflammation, fibrosis, pain, vasodilation, and smooth muscle contraction^5^. Several theories including developmental defects in the Tamm-Horsfall protein, potassium sensitivity theory, and autoimmunity have been posited. However their detail mechanisms remain unclear^6,7^.

IC can be classified into Hunner-type IC (HIC) and non-Hunner-type IC (NHIC). HIC is the classic, ulcerative type of IC and is characterized by patches of red mucosa with small vessels radiating from a central pale scar, known as “Hunner’s lesions”, on cystoscopy^8^. HIC is currently treated using transurethral resection and coagulation (TUR-C) with good outcomes^9^. Conversely, NHIC is characterized by glomerulations (multiple petechial-like hemorrhages) and submucosal haemorrhages without Hunner’s lesions on cystoscopy and hydrodistention, which is used as a therapeutic option and a diagnostic tool^10^.

We recently reported that NHIC is histopathologically associated with severe fibrosis and increased mast cell infiltration, and that HIC is associated with severe inflammation and urothelial denudation in the whole bladder^11^. Importantly, the severity of bladder tissue fibrosis in IC/BPS patients was associated with increased urinary frequency and decreased bladder capacity. Furthermore, our recent pre-clinical studies demonstrated the potential of that bladder tissue fibrosis as a promising therapeutic target for IC/BPS^12,13^. Beneficial outcomes have been obtained following anti-fibrotic approaches using *N*-acetylcysteine (NAC) or mesenchymal stem cell (MSC) therapy owing to the upregulation of WNT family genes, including *WNT2B*, *WNT5A*, *WNT8A*, *WNT8B*, *WNT10A*, and *WNT11*.

The WNT pathway is evolutionarily conserved and plays critical roles in embryonic and neonatal development. It is typically quiescent in several adult tissues^14^, but is reactivated in response to injury. The WNT pathway has complex and contrasting roles by promoting regeneration and fibrosis in several fibrotic disorders, such as renal, pulmonary, cardiac, and liver fibrosis^15–18^.

Few studies have investigated the association of fibrosis with WNT signalling cascades in IC/BPS pathogenesis. Hence, we evaluated this association and examined the functional role of dysregulated WNT genes in the bladder tissues of human IC/BPS patients.

## Results

A total of 30 patients were enrolled in the study with 25 IC patients (including 7 NHIC and 18 HIC), and 5 non-IC patients as a control. Patients were predominantly female (22 of 25 in the IC group and 4 of 5 in the control group). The mean age was 59.7 ± 10.9 years. The Pelvic Pain and Urgency/Frequency Patient Symptom Scale (PUF) scores, O’Leary-Sant Symptom and Problem Index (IC-Q) scores, and Visual Analogue Score (VAS) pain questionnaire scores did not differ between the NHIC and HIC groups. However, HIC patients demonstrated significantly shorter symptom duration, a higher urinary frequency, and smaller bladder capacity than NHIC patients (Table 1).

**Table 1:** Patient characteristics.

	Total (n = 30)	NHIC (n = 7)	HIC (n = 18)	p-value
Age (years)	59.7 ± 10.9	65.4 ± 7.3	56.5 ± 11.8	0.064
Gender (M/F)	4 vs 26	1 vs 6	2 vs 16	0.929
Duration of symptoms (months)	39.6 ± 31.9	70.8 ± 42.9	30.9 ± 22.8	0.046
PUF	22.6 ± 6.4	19.8 ± 4.0	23.7 ± 6.9	0.135
IC-Q	28.3 ± 7.0	25.0 ± 4.6	29.6 ± 7.5	0.051
VAS	6.9 ± 2.4	6.8 ± 1.7	7.0 ± 2.7	0.959
Voiding diary				
Urinary frequency (times/day)	13.3 ± 6.9	8.5 ± 2.6	16.1 ± 7.3	0.006
Urinary urgency (times/day)	9.5 ± 7.7	7.0 ± 3.5	10.9 ± 9.5	0.682
Urge incontinence (times/day)	0.6 ± 1.4	1.1 ± 1.6	0.4 ± 1.3	0.213
Maximum bladder capacity (ml)	262.6 ± 160.6	361.4 ± 133.6	208.6 ± 163.9	0.006

Standard abbreviations: EMT, epithelial-mesenchymal transition; GAG, Glycosaminoglycans; HIC, Hunner-type IC; IC/BPS, interstitial cystitis/bladder pain syndrome; IC-Q, O’Leary-Sant Symptom and Problem Index; MSCs, mesenchymal stem cells; NAC, *N*-acetylcysteine; NHIC, non-Hunner-type IC; PUF, Pelvic Pain and Urgency/Frequency Patient Symptom Scale; RQ-PCR, Real-time quantitative reverse transcription polymerase chain reaction; SHH, sonic hedgehog; TUR-C, transurethral resection and coagulation; VAS, Visual Analogue Score.

**Figure 1:**
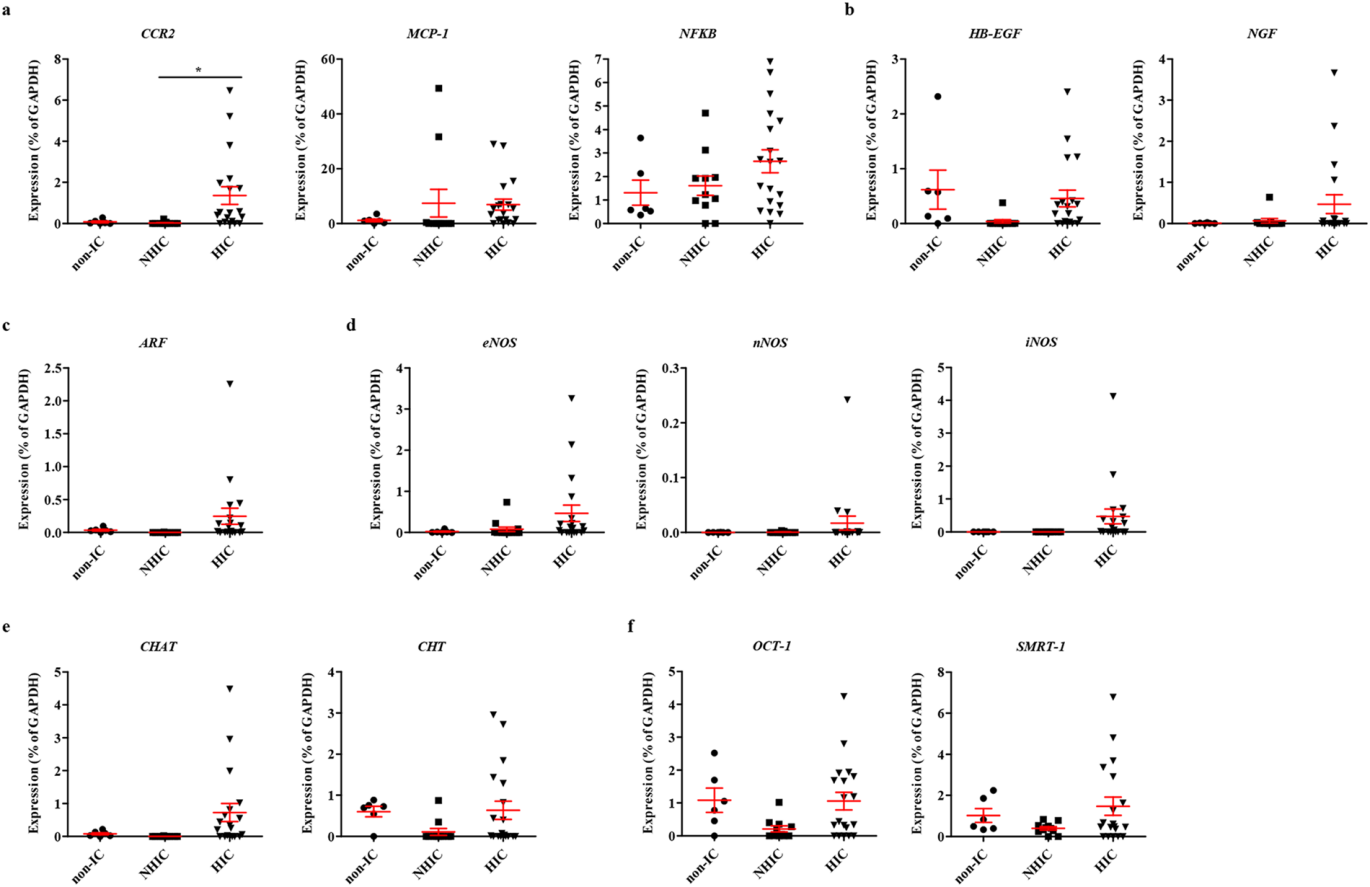
Comparison of gene expression associated with IC/BPS pathology between NHIC and HIC patient bladder tissues. **(a**–**f)** RQ-PCR analysis of the known biomarkers of IC/BPS, including those of inflammation **(a)**, growth factors **(b)**, apoptosis **(c)**, nitric oxide synthase **(d)**, acetylcholine neurotransmitter biosynthesis **(e)**, and transcription regulators **(f)** in the bladder tissues of the indicated patient groups. Gene expression is presented as a percentage of *GAPDH*. Data are represented as a dot plot of mean ± SEM [n = 6 for non-IC; n = 11 for non-Hunner-type IC (NHIC) and n = 19 for Hunner lesions in Hunner-type IC (HIC); *p < 0.05 using one-way ANOVA analysis with a Bonferroni post-hoc test]. Non-IC, stress urinary incontinence patients.

For gene expression assay, we employed the bladder biopsy specimens, which were confined to mucosa and submucosa layer of the bladder. We first examined the level of gene expression associated with IC/BPS pathogenesis including inflammation (e.g., *CCR2, MCP-1, NFκB*), growth factors (e.g., *HB-EGF, NGF*), nitric oxide synthase (e.g., *nNOS, iNOS, eNOS*), and apoptosis (e.g., *ARF*). Consistent with previous histological data^11^, the bladder tissues of HIC patients were characterised by the upregulation of pro-inflammatory genes, such as *CCR2* and *NFκB* (Fig. 1a), reflecting severe inflammation. The expression of *HB-EGF* and *NGF* was only slightly affected in the HIC group (Fig. 1b), despite urothelium denudation. Furthermore, in the bladder tissues of HIC patients, upregulation of *ARF*, which is associated with apoptosis (Fig. 1c), and nitric oxidase synthase (NOS) family genes, such as *iNOS*, *eNOS*, and *nNOS* (Fig. 1d), were observed although the difference was not statistically significant. Increased expression of choline O-acetyltransferase (*CHAT*), which is the biosynthetic enzyme for the neurotransmitter acetylcholine, was also observed (Fig. 1e). The bladder tissues of NHIC patients were characterised by downregulated expression of choline transporter (CHT, also known as solute carrier family 5 member 7; *SLC5A7*), (Fig. 1e) POU class 2 homeobox 1 (OCT-1), and nuclear receptor corepressor 2 (SMRT) transcription regulators (Fig. 1f). In comparison between control and IC/BPS (HIC and NHIC patients) groups, the difference in the expressions of these genes were not statistically significant (Supplementary Fig. 1). Thus, these findings demonstrate that HIC and NHIC have distinct gene expression patterns, as observed for histological profiles^11^.

**Figure 2:**
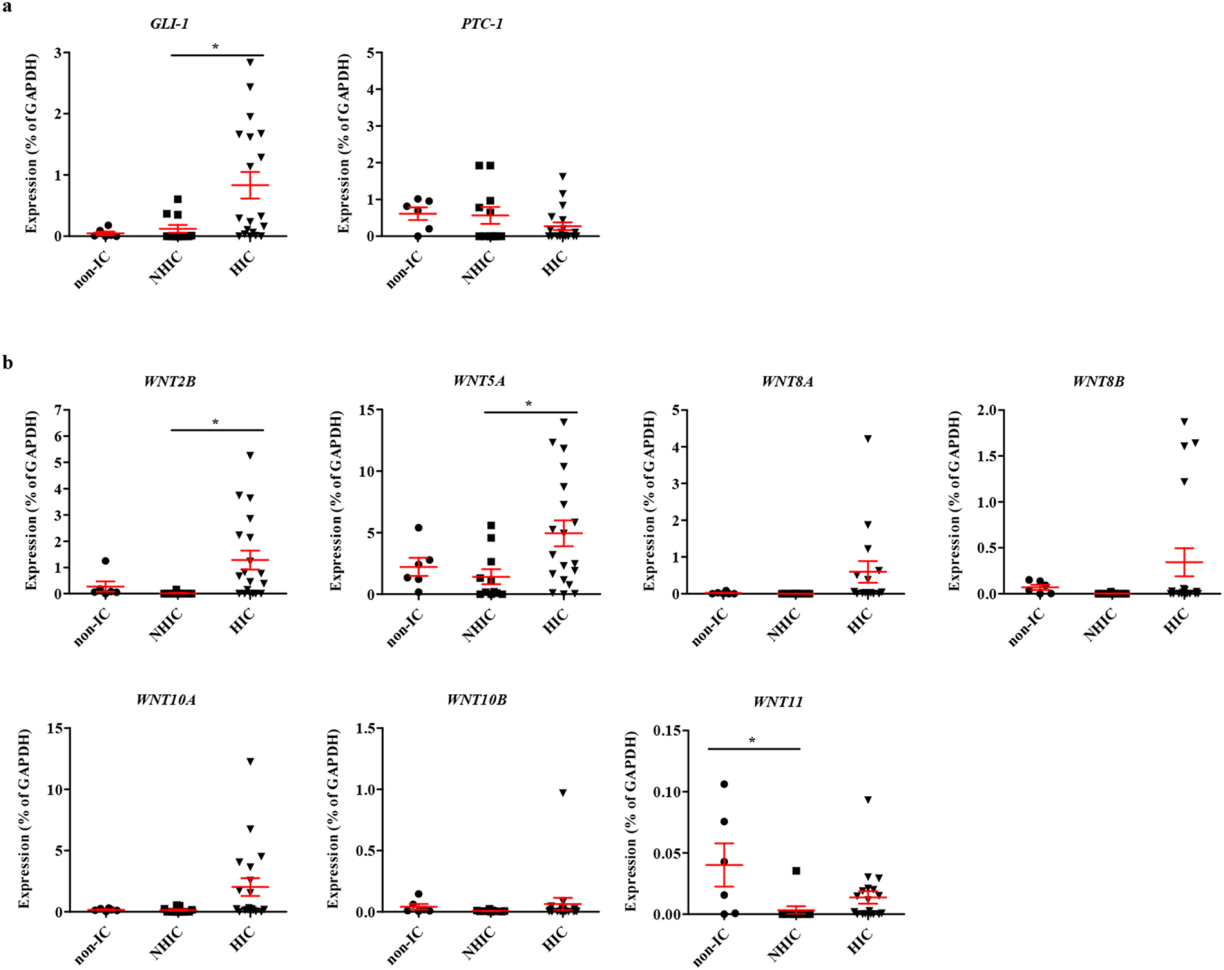
Downregulation of SHH or WNT family genes in the bladder tissue of NHIC patients. **(a**,**b)** RQ-PCR analysis of the genes involved in SHH **(a)** and WNT **(b)** pathways in the bladder tissues of the indicated patient groups. Gene expression is presented as percentage of *GAPDH*. Data are represented as a dot plot of mean ± SEM [n = 6 for non-IC; n = 11 for NHIC and 19 for HIC patient groups; *p < 0.05 by one-way ANOVA analysis with a Bonferroni post-hoc test].

We recently reported that the SHH and WNT gene families are particularly responsible for the mechanisms underlying MSC therapy in IC/BPS animal models^12,13^. Thus, changes in the expression of these genes was examined in the bladder tissues of IC/BPS patients, focusing on the genes validated in pre-clinical MSC studies. Expression of the SHH pathway mediator *GLI-1* was upregulated in the HIC patient group. However, the expression of *PTC-1*, a transmembrane receptor binding to SHH, was only slightly changed (Fig. 2a). Among the WNT family genes, *WNT2B* and *WNT5A* transcripts were increased only in the bladder tissues of the HIC patients (Fig. 2b). In particular, *WNT11* expression was significantly down-regulated in the bladder tissues of IC/BPS patients (Supplementary Fig. 2), and the repression of WNT11 was characteristically observed in the NHIC patients (Fig. 2b). Overall, WNT gene expression in the bladder tissues was lower in the NHIC patients than in the HIC patients and WNT11 expression was significantly repressed in the NHIC patients (Fig. 2b).

**Figure 3:**
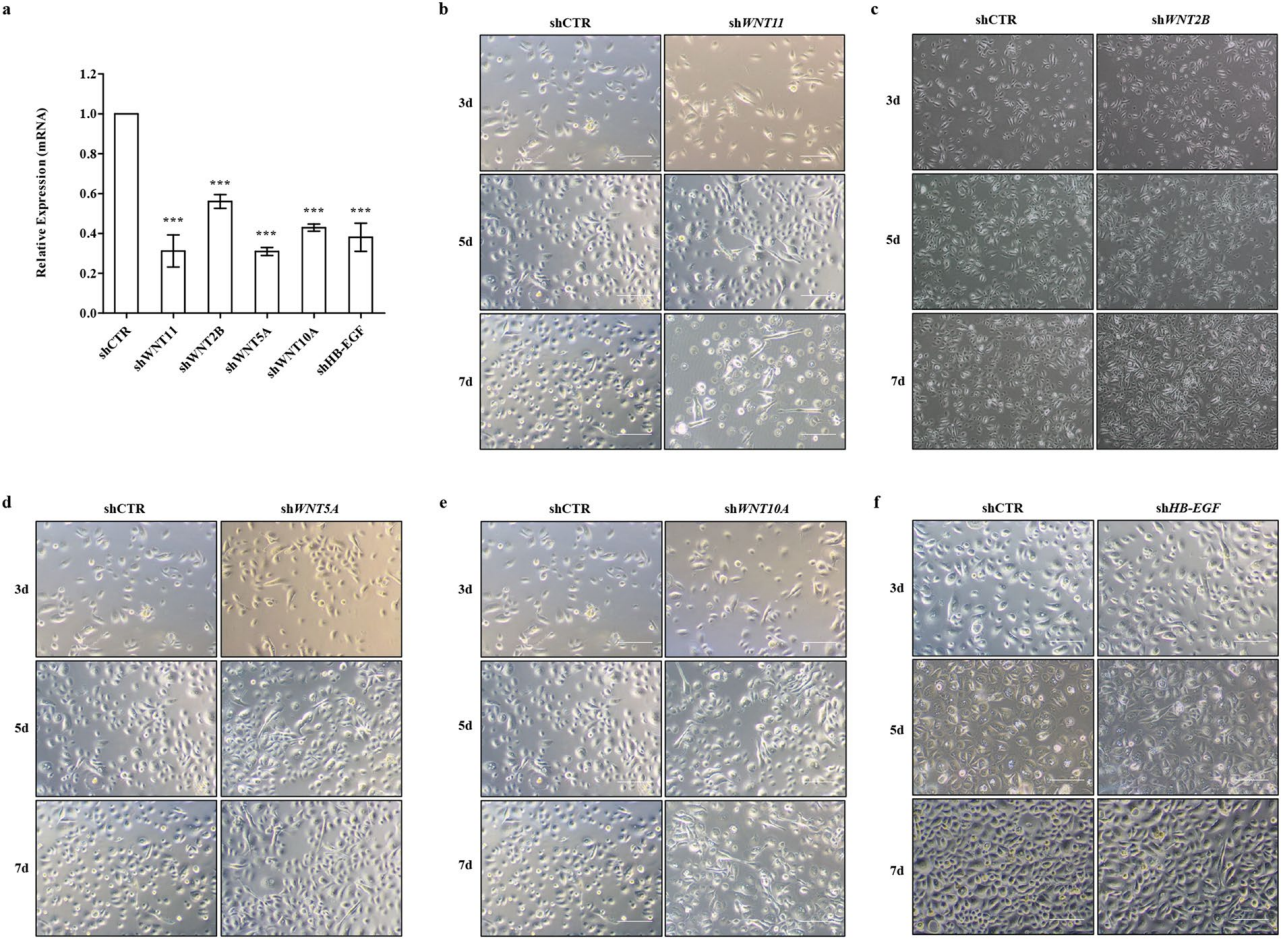
Morphological changes following silencing of WNT family genes in HBlEpC. **(a)** RQ-PCR analysis of the indicated genes in HBlEpC primary bladder epithelial cells at 4 days after infection with lentivirus containing each shRNA construct. Data normalised to the scramble control group (shCTR) are represented as mean ± SEM, n = 4, ***p < 0.001 using one-way ANOVA analysis. **(b**–**f)** Representative image of HBlEpC at the indicated days after infection with lentivirus containing sh*WNT11* **(b)**, sh*WNT2B* **(c)**, sh*WNT5A* **(d)**, sh*WNT10A* **(e)**, and sh*HB-EGF* **(f)** or scramble control (shCTR) construct (magnification x100, scale bar = 100 μm).

**Figure 4:**
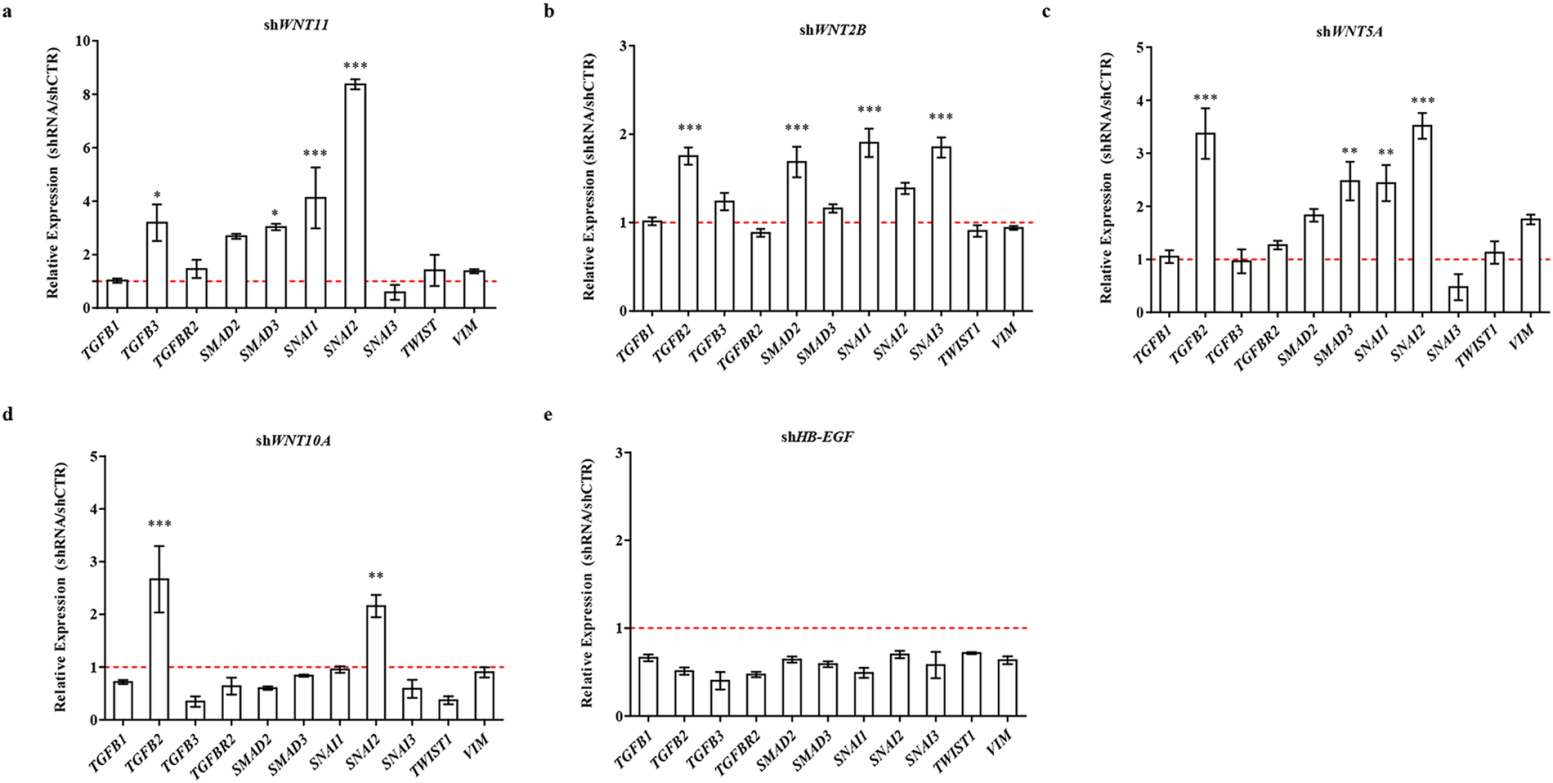
Upregulation of fibrosis-related genes by knockdown of WNT family genes. **(a**–**e)** RQ-PCR analysis of genes involved in TGFβ signalling (e.g., *TGFB1*, *TGFB2*, *TGFB3*, *TGFBR2*, *SMAD2*, and *SMAD3*) and EMT (e.g., *SANI1*, *SNAI2*, *SNAI3*, *TWIST1*, and *VIM*) in HBlEpC cells at 7 days after infection with lentivirus containing sh*WNT11* **(a)**, sh*WNT2B* **(b)**, sh*WNT5A* **(c)**, sh*WNT10A* **(d)**, and sh*HB-EGF* **(e)** constructs. A scramble construct was used as the control (shCTR). Gene expression is presented as expression relative to the shCTR and is represented as mean ± SEM, n = 4, *p < 0.05, **p < 0.01, ***p < 0.001 using one-way ANOVA analysis.

**Figure 5:**
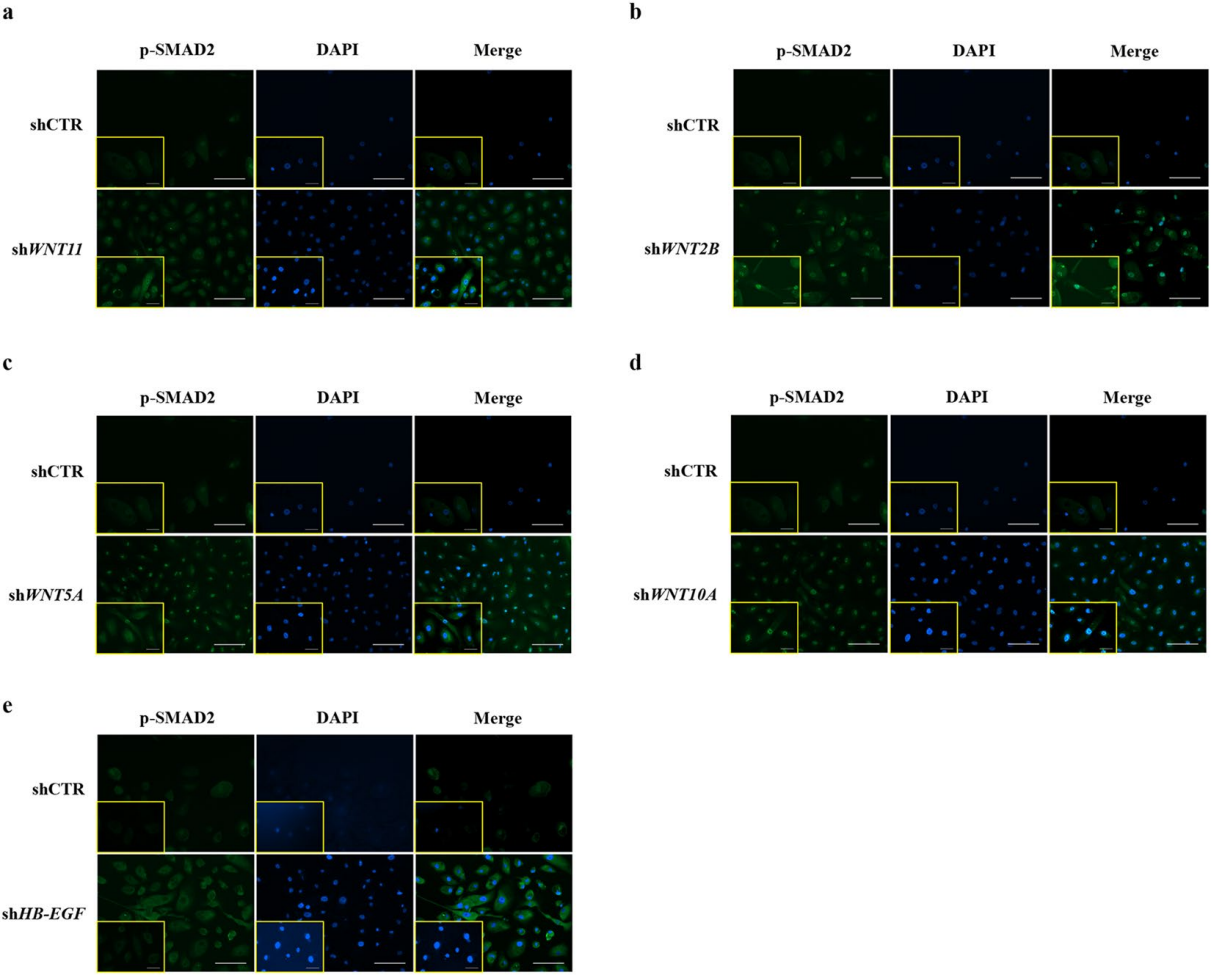
Nuclear localization of phosphorylated SMAD2 protein induced by downregulation of WNT family genes. **(a**–**e)** Immunofluorescent staining for nuclear localization of phosphorylated SMAD2 protein (green) in HBlEpC cells at 4 days after infection with lentivirus containing sh*WNT11* **(a)**, sh*WNT2B* **(b)**, sh*WNT5A* **(c)**, sh*WNT10A* **(d)**, and sh*HB-EGF* **(e)** constructs (magnification x200, scale bar = 100 μm). The higher magnification images (magnification x400, scale bar = 50 μm) are inserted in lower left corner in each image. Nuclei were stained with DAPI (blue). A scramble shRNA construct was used as the control.

**Figure 6:**
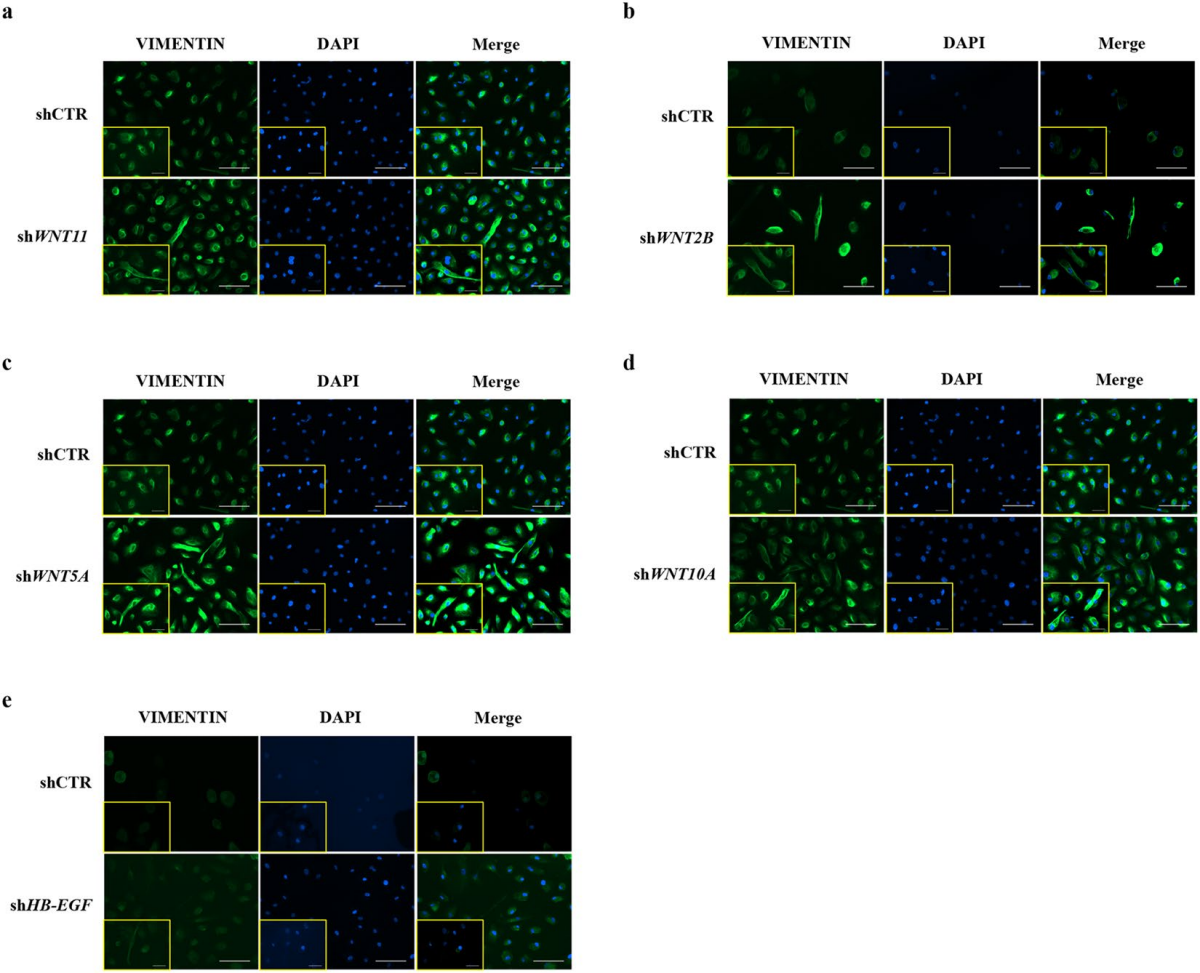
Induction of vimentin intermediate filament protein by silencing of WNT family genes. **(a**–**e)** Immunofluorescent staining of vimentin (green), an intermediate filament protein induced by the EMT process in HBlEpC cells at 4 days after infection with lentivirus containing sh*WNT11* **(a)**, sh*WNT2B* **(b)**, sh*WNT5A* **(c)**, sh*WNT10A* **(d)**, and sh*HB-EGF* **(e**) constructs (magnification x200, scale bar = 100 μm). The higher magnification images (magnification x400, scale bar = 50 μm) are inserted in lower left corner in each image. Nuclei were stained with DAPI (blue). Note that low vimentin expression was observed following downregulation of *HB-EGF* unlike that following downregulation of WNT family genes. A scramble shRNA construct was used as the control.

**Figure 7:**
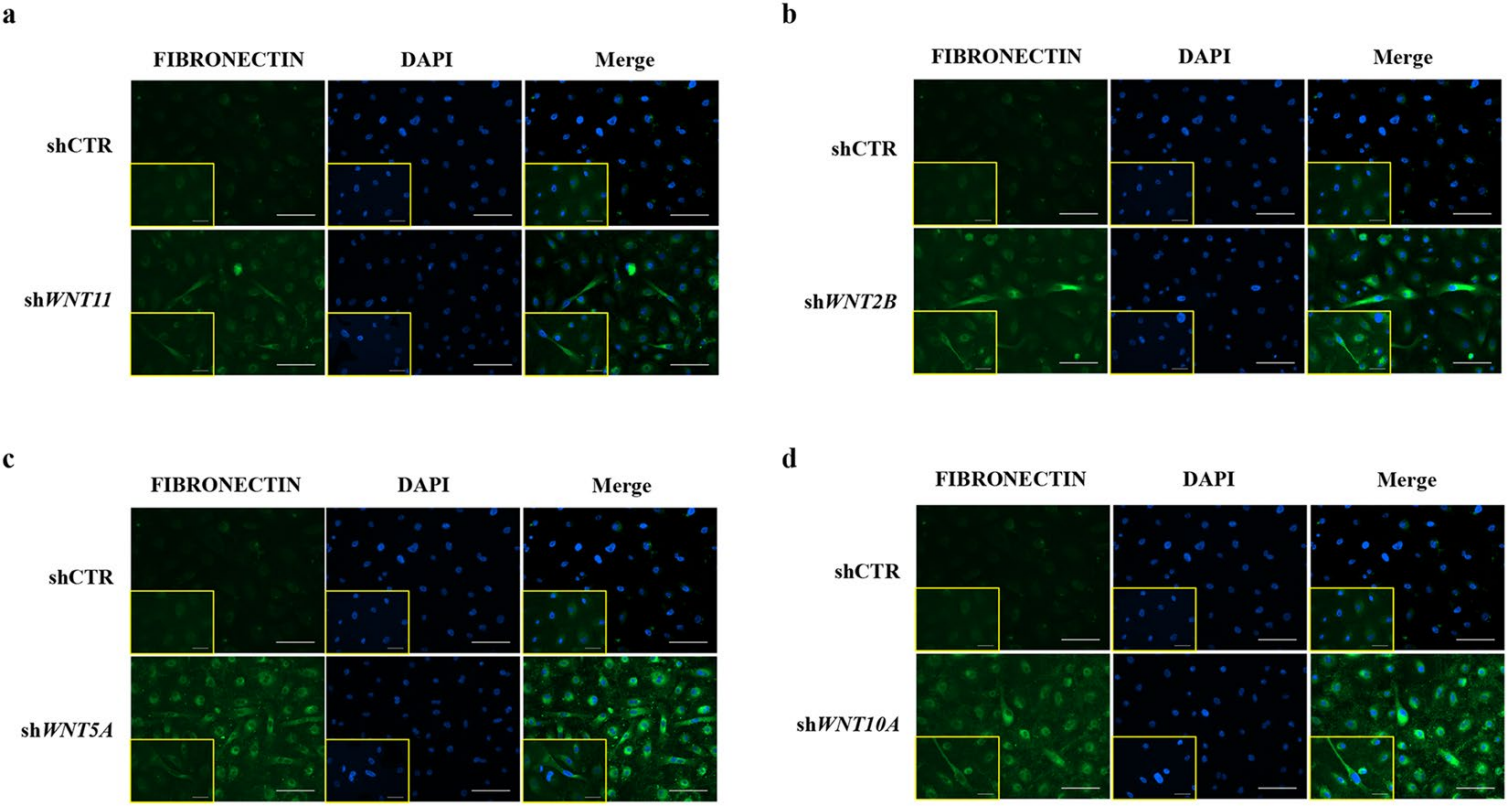
Increased fibronectin extracellular matrix protein by knockdown of WNT family genes. **(a**–**d)** Immunofluorescent staining of fibronectin (green), an extracellular matrix protein in HBlEpC cells, at 7 days after infection with lentivirus containing sh*WNT11* **(a)**, sh*WNT2B* **(b)**, sh*WNT5A* **(c)**, and sh*WNT10A* **(d)** constructs (magnification x200, scale bar = 100 μm). The higher magnification images (magnification x400, scale bar = 50 μm) are inserted in the lower left corner in each image. Nuclei were stained with DAPI (blue). A scramble shRNA construct was used as the control.

**Figure 8:**
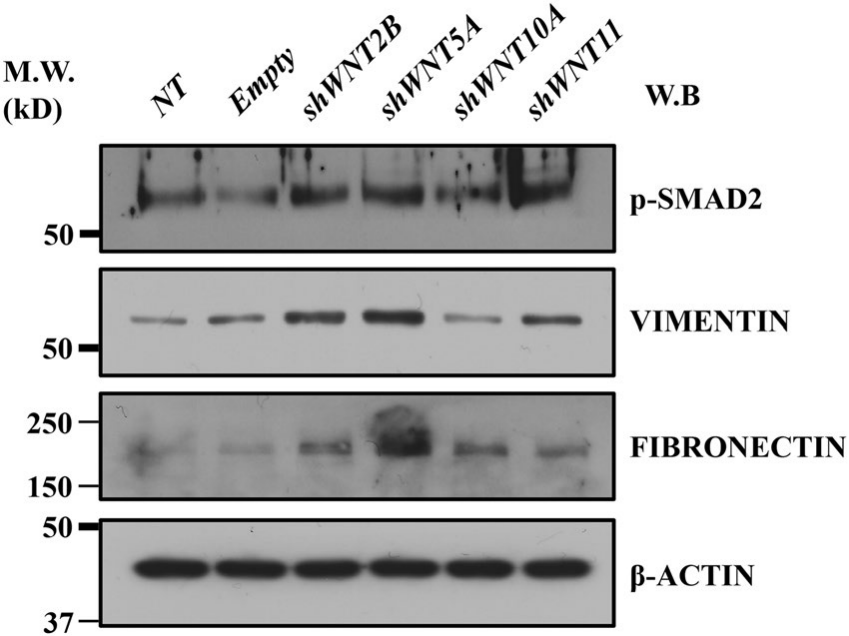
Western blot analysis of TGFβ activation and fibrosis by knockdown of WNT family genes. Western blot was used to detect the phosphorylated SMAD2 protein (p-SMAD2), vimentin, and fibronectin proteins in HBlEpC cells at 7 days after infection with lentivirus containing sh*WNT2B*, sh*WNT5A*, sh*WNT10A*, and *shWNT11* constructs. For control, HBlEpC cells without infection (not-treated; NT) or infected with empty lentivirus (Empty) were used. β-ACTIN was used as a loading control. Molecular weight (M.W.) marker sizes (kD) are shown on the left.

We further characterised the potential effects of WNT pathway dysregulation by infecting HBlEpC with lentivirus containing specific short hairpin (sh)RNA, thus silencing every WNT family gene that was altered in IC/BPS patients (Fig. 3a). For comparison, knockdown of *HB-EGF*, a growth factor responsible for urothelium integrity, was also performed. Silencing of *WNT11* induced fibrosis which was microscopically evident (Fig. 3b). The induction of fibrotic change was also observed by knock-down of *WNT2B*, *WNT5A*, and *WNT10A*, which were significantly up-regulated in the bladder tissues of HIC patients (Fig. 3c–e). Conversely, these fibrotic changes were barely observed in *HB-EGF* knockdown in HBlEpC cells (Fig. 3f). Consistent with these results, silencing of these WNT genes, unlike *HB-EGF*, activated the genes associated with transforming growth factor-beta (TGF-β) signalling, such as *TGFB2*, *SMAD2*, and *SMAD3* and epithelial-mesenchymal transition (EMT), such as *SNAI1, SNAI2, TWIST*, and vimentin (*VIM*) (Fig. 4)^19^. Activation of TGF-β signalling in these WNT silenced HBlEpC cells was further validated by immunostaining for nuclear localisation of phosphorylated SMAD2 (Fig. 5a–d) and increased level of vimentin, a cytoskeletal protein (Fig. 6a–d). Conversely, silencing of *HB-EGF* only slightly affected TGF-β signalling (Figs 5e and 6e). Accordingly, downregulation of these WNT genes resulted in an increased expression of fibronectin, an extracellular matrix protein (Fig. 7). The activation of TGF-β signalling and increased proteins associated with fibrosis in the WNT knock-downed HBlEpC cells were further validated by Western blot assay (Fig. 8). Taken together, these *in vitro* functional studies indicated that mis-regulation of a subset of WNT genes including *WNT11* could trigger fibrotic changes in the epithelial cells of the bladder.

## Discussion

Here, we observed that downregulation of *WNT11* is associated with bladder tissue fibrosis in NHIC patients than in HIC patients. Moreover, silencing of WNT family genes in bladder epithelial cells induced fibrotic changes. Higher *WNT2B* and *WNT5A* expression was observed in HIC patients than in NHIC patients. *WNT11* expression was characteristically down-regulated in NHIC patients. To our knowledge, this is the first study to investigate the relationship between the WNT signalling pathway and bladder tissue fibrosis in IC/BPS patients.

IC is classified into HIC and NHIC. However, whether they belong to the same disease entity is still controversial. In practice, the phenotypes of both types of IC differ. A diagnosis of HIC can be established based on the presence of Hunner’s lesions. However, such lesions are absent in NHIC patients. Therefore, urine or tissue biomarkers may be helpful for the diagnosis of IC. Previously, we observed that NHIC is characterised by severe fibrosis^11^. Thus, we attempted to identify genes that are associated with fibrosis in NHIC patients.

The WNT gene functions in embryonic development, and acts as an oncogene when aberrantly triggered^20^. In our previous study, we revealed that WNT signalling genes were downregulated in the IC rat model^13^. The WNT signalling pathway is known to modulate tissue fibrosis and associated EMT processes^21^. Thus, we further investigated the association of fibrosis with *WNT* signalling cascades in IC/BPS patients.

In this study, *WNT11* expression was remarkably repressed in NHIC patients. In our previous study, we observed that NHIC was characterised by severe fibrosis and increased mast cell infiltration, while HIC was characterised by severe inflammation and urothelial denudation in the whole bladder^11^. These results indicate that the downregulation of *WNT11* enhances EMT activation and bladder tissue fibrosis, which is mainly observed in an NHIC bladder. Thus, we propose that inhibition of the *WNT11* would be novel pathogenesis and differential diagnosis of NHIC.

In our functional study using HBlEpC, we selected *WNT2B*, *WNT5A*, and *WNT11*, as their expression is lower in NHIC patients, and selected *WNT10A* as its expression is lower in the NHIC group than in the HIC group, although there was no clinical significance. *HB-EGF*, a growth factor responsible for urothelial integrity is a potential biomarker for the diagnosis of IC/BPS. Further, its expression is higher in NHIC patients than in HIC and non-IC patients. Hence, it was selected for comparison^22^. The knockdown of all selected WNT genes induced fibrotic changes in EMT-related morphology and activated the TGF-β pathway (Figs 4–8). However, these fibrotic changes were barely observed in *HB-EGF*-silenced HBlEpCs. These *in vitro* functional studies support our hypothesis that downregulation of WNT genes triggers fibrotic changes in bladder epithelial cells, thus playing a pivotal role in the pathogenesis of NHIC.

Studies on IC/BPS have been published for a century, but pathophysiology of IC/BPS is still complex and multifactorial. Numerous reports have implicated mast cell activation, GAG layer defects, developmental defects in the Tamm-Horsfall protein, and the autoimmune theory in the pathophysiology of IC/BPS. However, consensus is lacking^2–4,7,23^. Studies have proposed antiproliferative factor, epidermal growth factor, GAGs, bladder nitric oxide, and multiple urine proteins and serum cytokines as potential biomarkers of IC/BPS^24^. However, no definite biomarker has been identified for the differential diagnosis of IC/BPS. Furthermore, to our knowledge few studies on biomarkers of NHIC have been conducted, whereas urinary CXCL10 has been recently reported as a promising biomarker and the Bladder Permeability Defect Risk Score (BP-RS) using crowdsourcing has been developed (IP4IC study) in HIC patients^25,26^. Of note, no study has reported that downregulation of WNT pathway leads to fibrotic changes of HBlEpCs, and severe fibrosis observed in NHIC is strongly associated with decreased levels of WNT-related gene expression.

In this study, HIC patients had significantly more episodes of urinary frequency (16.1 versus 8.5 times/day, p = 0.006) and lower maximal bladder capacity (208.6 ml versus 361.4 ml, p = 0.006) than NHIC patients. Duration of symptom was significantly shorter (30.9 vs 70.8 months, p = 0.046) in HIC patients. This is probably due to the severity in symptoms, which might have provoked patients to seek earlier medical treatment leading to earlier diagnosis. Conversely, as the symptoms are less severe in NHIC, diagnosis and treatment could be delayed. Therefore, it is important to identify biomarkers that can help diagnose NHIC quickly.

Patients can be easily diagnosed with HIC owing to characteristic symptoms, such as bladder pain and/or urinary urgency/frequency, and Hunner’s lesions observed on cystoscopy. However, if Hunner’s lesions is not observed on cystoscopy and obvious glomerulation or submucosal haemorrhage is not observed during hydrodistention, a diagnosis of NHIC will be difficult to establish. Thus, histopathologic and gene expression studies of a bladder mucosal biopsy sample can aid in the differential diagnosis of IC/BPS by identifying fibrotic changes and WNT-related gene expression levels. Fibrotic changes could be a potential therapeutic target for NHIC. Indeed, in the rat models in our previous study, direct administration of MSCs into the submucosal layer of the bladder significantly restored voiding function and ameliorated tissue fibrosis^12,13^. These preclinical results indicate that fibrosis of the bladder epithelium may be treatable and require additional clinical trials using stem cells for confirmation. The use of antifibrotic agents, such as NAC, which was effective in an IC rat model, may be helpful for treating fibrosis in NHIC patients^27^.

This study has several limitations. First, the biopsy specimens in this study were confined to the mucosa and submucosa layer of the bladder and *in vitro* functional assays of WNT genes were investigated using only primary bladder epithelial cells. In this regard, the results represent the pathogenesis, which could be responsible mainly for the urothelium, but not the whole bladder. Further studies employing the animal models with the alternation of the WNT genes in entire bladder tissues could be required to understand the precise role of these WNT genes on the pathogenesis of IC/BPS. Second, this study involves a tissue biomarker and not a urine biomarker, and although urine-based studies are difficult, a urine biomarker is ideal because urine collection is more convenient and noninvasive than a bladder mucosal biopsy. Third, the sample size of each group was heterogeneous. The number of patients was relatively smaller in the NHIC and control groups than in the HIC group; hence, it was difficult to obtain significant differences between each group. A prospective study involving a higher number of participants is required in the future. Nevertheless, novel findings have been obtained here via the WNT signalling pathway, which have not been shown in previous IC/BPS studies.

In conclusion, downregulated WNT genes resulted in fibrotic changes in bladder epithelial cells and is significantly associated with the pathogenesis and differential diagnosis of NHIC. Decreased expression WNT genes could have diagnostic value as a biomarker for predicting NHIC. Additional clinical trials using stem cells or antifibrotic agents could be helpful for treating IC/BPS patients.

## Methods

### Study approval

This prospective study was approved by the Institutional Review Board of Asan Medical Center and was conducted in accordance with the Declaration of Helsinki. Informed consent was obtained from all patients prior to enrollment in the study.

### Human samples

Diagnosis of IC/BPS was based on the American Urological Association criteria^11^. Patients’ baseline symptoms were assessed with the IC-Q, PUF, and VAS pain questionnaire. Inclusion criteria was ≥13 points on the PUF, ≥12 points on the IC-Q with ≥2 points on the pain and nocturia categories, and ≥4 points on the VAS. Thorough history taking, physical examination, urine culture, urine cytology, cystoscopy, and abdominopelvic computed tomography (CT) were performed at the outpatient clinic to specify the subtypes of IC, and to exclude patients with active urinary tract infections, urological malignancies, urolithiasis, neurologic diseases and pathologic pelvic conditions, such as malignancies or endometriosis. For the control group (non-IC), five patients receiving a sling operation for stress urinary incontinence with preoperative microscopic haematuria were enrolled.

Based on the presence of Hunner’s lesion on preoperative cystoscopy, we divided patients into NHIC (negative H-lesion) and HIC (positive H-lesion) group. NHIC patients were treated with hydrodistension of the bladder and HIC patients were treated with TUR-C. In the NHIC group, bladder biopsy was performed after hydrodistension. In the control group, biopsy was performed after the sling operation, while identifying the reason for microscopic haematuria and injury in bladder wall or urethra. In both groups, we randomly targeted three sites on the posterior wall, both lateral walls of the bladder and cold cup biopsy was done with biopsy forceps. In HIC patients, Hunner’s lesion was targeted and specimens were obtained with endoscopic electrosurgical loop. Unlike routine TUR of bladder tumour, resection depth was confined to mucosa and submucosa layer of the bladder. Muscularis propria was not included in TUR specimen. Similarly, cold cup biopsy specimens included mucosa and submucosa layer of the bladder.

### Real-time quantitative reverse transcription polymerase chain reaction (RQ-PCR)

Expression of the WNT pathway genes and the pathology of IC/BPS were examined using RQ-PCR, as previously described^28,29^. Briefly, bladder tissues were freshly obtained from patients and total RNA was extracted using the RNeasy Mini Kit (QIAGEN, Valencia, CA). The RNA was treated with DNase I (QIAGEN) to remove contaminated genomic DNA. Total RNA (400 ng) was reverse-transcribed using TaqMan Reverse-Transcription Reagents (Applied Biosystems, Waltham, MA), according to the manufacturer’s instructions. Target gene expression was quantified via RQ-PCR with the HOT FIREPol® EvaGreen® qPCR Mix Plus (Solis BioDyne, Tartu, Estonia) on the PikoReal™ Real-Time PCR System (Thermo Fisher Scientific, Pittsburgh, PA). The threshold cycle (*C*t) and the cycle number at which the fluorescence of the amplified gene reaches a fixed threshold were subsequently determined, and the relative expression level of the target genes was quantified via the 2^−ΔΔ^Ct method using *glyceralydehyde-3-phosphate dehydrogenase* mRNA levels as an endogenous control and the mRNA levels of the indicated groups as calibrators. All primers used for RQ-PCR analysis are listed in Table 2.

**Table 2:** Sequences of the primers used in this study.

Gene	Symbol	Forward Primer	Reverse primer
C-C motif chemokine receptor 2	CCR2	TACGCTCCATCGCTGTCATCTC	GCGAAGCACTGAAACACTCGAA
C-C motif chemokine ligand 2	MCP-1	TTCCCCTAGCTTTCCCCAGA	TCCCAGGGGTAGAACTGTGGT
nuclear factor kappa B subunit 1	NFKB	TGC ATC TGG GGA TGA GGT TG	TGG TCA GAA GGA ATG CCA GG
heparin binding EGF like growth factor	HB-EGF	CAA GTC TCA GAA GAG GTT GGG C	CAC CAG AAG AAT GGC AGG AGT T
nerve growth factor	NGF	AAG CGG TCA TCA TCC CAT CC	CAC CTC CTT GCC CTT GAT GTC
nitric oxide synthase 1	nNOS	ATC CAG TGC TCT TGA GCT GGG	TTG GGC CTT CTG GAA AAC CA
nitric oxide synthase 2	iNOS	TCG GAG CCT CCT CTC TCA AAC T	GGT GCA CTC AGC AGC AAG TTC
nitric oxide synthase 3	eNOS	TGG GTC CGC CTT AAT CTG G	TGT AAT CCA CAT GAG CTG GGG
cyclin dependent kinase inhibitor 2A	ARF	GAG GCT CTG AGA AAC CTC GGG	AAA ACT ACG AAA GCG GGG TGG
choline O-acetyltransferase	CHAT	ATG GCC ATT GAC AAC CAC CTG	GCA GCA GAA CAT CTC CGT GGT
solute carrier family 5 member 7	CHT	TTG TAC CCA TCA TGT GCT CTG TT	TAT CCA CAG GTG TTG CCT TCC
jumping translocation breakpoint	PAR	TGG AAG TTC GAA GGG GCT G	CTC GAT TTG CTT CCG GAC CT
POU class 2 homeobox 1	OCT_1	AGC AGC TTG AAT GAG GCA GTG	TTT GGC TAA CAG GCA CTC TGG
nuclear receptor corepressor 2	SMRT	TTA GCG CTC TGG ACA GAT GGA	TGG CCT GAC TTG GTT TCC AG
GLI transcription factor family	GLI-1	CCA GCT GTG GTC ATC CTG AGG	AAG ATC AAG AGA GTC CAG GGG GTT
patched 1	PTC-1	AGT TAC CAT TGG CGA CCT AGC AT	TGA TGG CTC CAA CAC TAA CTG TCT C
Wnt family member 2B	WNT2B	CTC CCT GAT TTC CCG CTC TG	AGA AGT ATC GGG AAG CTG GTG C
Wnt family member 5A	WNT5A	AAT AGG CAC GAA GGC ACA GGT C	AAC ACG GCA TCT CTC TTT CAC CA
Wnt family member 4	WNT4	CAT CCT GCC CAA ACC ACT CTC	CGT CAC AAT GGC AAA GAG CC
Wnt family member 8A	WNT8A	GCA GAG GCG GAA CTG ATC TTT	GTT GTG GCT GTT CTG TAG GCA CT
Wnt family member 8B	WNT8B	GGG GTT GGT TCC TAG AGG CAG	TGT ATC TGG AGT CCC TCG GGT T
Wnt family member 11	WNT11	TCT TTG GGG TGG CAC TTC TC	TCT GCC GAG TTC ACT TGA CG
Wnt family member 10A	WNT10A	TTG GCT CTT GGG AAG AGG AGA	TGA GTG GTG GGG TTC AGA CAG
Wnt family member 10B	WNT10B	CCA GGC CCT TAG GGA AGT TG	CCA CCC TTC CTG CTG AAG AA

### Cell culture

HBlEpC, a primary epithelial cell line derived from the normal human bladder (Cell Applications, Inc, San Diego, CA) was maintained in Bladder Epithelial Cell Growth Medium (Cell Applications, Inc), according to the manufacturer’s instructions.

### Gene knockdown

For RNA interference mediated gene silencing, shRNA constructs were designed to target 19 base-pair gene-specific regions of *WNT2B*, *WNT5A*, *WNT10A*, or *HB-EGF* and then then cloned into a pLenti6/BLOCK-iT™-DEST lentiviral vector using the Gateway Technology reaction as previously described^28,30^. The lentivirus was generated using a four-plasmid transfection system (Invitrogen, Waltham, MA). Two days after transfection into the 293 FT packaging cell line, supernatants containing recombinant pseudo-lentiviral particles were collected and concentrated by precipitation using Lenti-X Concentrator kit (Clontech, Mountain View, CA) according to the manufacturer’s instructions (Invitrogen). HBlEpC cells were infected with the concentrated virus using 6 μg/ml polybrene (Invitrogen). The effect of gene knockdown was examined using RT-PCR 5 days after infection. The sequences for the top and bottom oligonucleotides for each shRNA are listed in Table 3.

**Table 3:** Sequences of the top and bottom oligonucleotides for each shRNA used in this study.

*WNT2B*_shRNA_ Top	**CAC CGC ACG AGT GAT CTG TGA CAA TCG AAA TTG TCA CAG ATC ACT CGT GC**
*WNT2B*_shRNA_ Bottom	**AAA AGC ACG AGT GAT CTG TGA CAA TTT CGA TTG TCA CAG ATC ACT CGT GC**
*WNT5A*_ shRNA_Top	CAC CGA AGT GCA ATG TCT TCC AAG TCG AAA CTT GGA AGA CAT TGC ACT TC
*WNT5A*_ shRNA_ Bottom	AAA AGA AGT GCA ATG TCT TCC AAG TTT CGA CTT GGA AGA CAT TGC ACT TC
*WNT5A*_ shRNA_Top	CAC CGC AAG TTG GTA CAG GTC AAC ACG AAT GTT GAC CTG TAC CAA CTT GC
*WNT5A*_ shRNA_Top	AAA AGC AAG TTG GTA CAG GTC AAC ATT CGT GTT GAC CTG TAC CAA CTT GC
*WNT10A* _shRNA_ Top	**CAC CGA GAC ATC CAC GCG AGA ATG ACG AAT CAT TCT CGC GTG GAT GTC TC**
*WNT10A*_ shRNA_ Bottom	**AAA AGA GAC ATC CAC GCG AGA ATG ATT CGT CAT TCT CGC GTG GAT GTC TC**
*WNT11*_shRNA_ Top	CAC CGC GTG TGC TAT GGC ATC AAG TCG AAA CTT GAT GCC ATA GCA CAC GC
*WNT11*_ shRNA_ Bottom	AAA AGC GTG TGC TAT GGC ATC AAG TTT CGA CTT GAT GCC ATA GCA CAC GC
*HB-EGF*_shRNA_ Top	**CAC CGA AAG TCC GTG ACT TGC AAG ACG AAT CTT GCA AGT CAC GGA CTT TC**
*HB-EGF*_ shRNA_ Bottom	**AAA AGA AAG TCC GTG ACT TGC AAG ATT CGT CTT GCA AGT CAC GGA CTT TC**
*HB-EGF*_shRNA_ Top	**CAC CGG GAC CCA TGT CTT CGG AAA TCG AAA TTT CCG AAG ACA TGG GTC CC**
*HB-EGF*_ shRNA_ Bottom	**AAA AGG GAC CCA TGT CTT CGG AAA TTT CGA TTT CCG AAG ACA TGG GTC CC**

### Immunocytochemistry and western blot analysis

HBlEpC cells cultured on glass coverslips were fixed with 4% paraformaldehyde, and permeabilised with 0.1% Triton X-100. Cells were blocked with 1% bovine serum albumin in phosphate buffer solution for 30 min at room temperature and subsequently stained with antibodies against Phospho-Smad2 (#3101; Cell Signaling Technology, Danvers, MA), vimentin (sc-6260; Santa Cruz Biotechnology, Santa Cruz, CA), and fibronectin (sc-8422; Santa Cruz Biotechnology). Immunostaining was visualized using Alexa 488 (A11001) or Alexa 488 (A11008)-conjugated anti-mouse or rabbit antibodies (Molecular Probes, Grand Island, NY). Nuclei were counterstained with 4,6-diamidino-2-phenylindole (D9542; DAPI, Sigma-Aldrich). Images were obtained using a fluorescence microscope (EVOS® FL Imaging System, Life Technologies Microscopy, Carlsbad, CA).

For western blot analysis, cell extracts (30 μg) were prepared in RIPA lysis buffer (Santa Cruz Biotechnology) and separated on 12% SDS-PAGE gels. The expression level of the indicated proteins was assessed by probing with same antibodies used in immunocytochemistry assay. For loading control, β-actin (A5441; Sigma-Aldrich) was used. Uncropped western blot images were found in Supplementary Fig. 3.

### Statistics

Data were analysed using GraphPad Prism 6.0 software (GraphPad Software, La Jolla, CA) and are expressed as mean ± standard error of the mean (SEM). The differences and significance were verified using one-way ANOVA followed by Bonferroni post hoc tests. A p-value < 0.05 was considered statistically significant.

## Electronic supplementary material

Supplementary Information
