# Supplementary material for: Downregulation of WNT11 is associated with bladder tissue fibrosis in patients with interstitial cystitis/bladder pain syndrome without Hunner lesion
Source: Sci Rep. 2018 Jun 28;8:9782. doi: 10.1038/s41598-018-28093-7 (PMC6023880; doi:10.1038/s41598-018-28093-7)
Supplement: Supplementary file 1 — Supplementary Information [file 41598_2018_28093_MOESM1_ESM.pdf]

Supplementary Information

Downregulation of *WNT11* is associated with bladder tissue fibrosis in patients with interstitial cystitis/bladder pain syndrome without Hunner lesion

Daeheon Choi[1*], Ju-Young Han[1,2,*], Jung Hyun Shin[1], Chae-Min Ryu[1,2], Hwan Yeul Yu[1,2], Aram Kim[4], Seungun Lee[2,3], Jisun Lim[2,3], Dong-Myung Shin[2,3,†], Myung-Soo Choo[1,†]

[1]Department of Urology, [2]Department of Biomedical Sciences, [3]Department of Physiology

Asan Medical Center, AMIST, University of Ulsan College of Medicine, Seoul, Korea

[4]Department of Urology, Konkuk University Hospital, Konkuk University School of Medicine, Seoul, Korea

*These authors equally contributed to this work.

[*]Correspondence:

Myung-Soo Choo, M.D., Ph.D, Department of Urology, Asan Medical Center, University of Ulsan College of Medicine, 88 Olympic-ro 43-gil, Songpa-gu, Seoul 05505, Korea

Tel: +82-2-3010-3735; Fax: +82-2-477-8928; Email: mschoo@amc.seoul.kr

Dong-Myung Shin, Ph.D., Department of Biomedical Sciences, Asan Medical Center, University of Ulsan College of Medicine, 88 Olympic-ro 43-gil, Songpa-gu, Seoul 05505, Korea

Tel: +82-2-3010-2086; Fax: +82-2-3010-8493; Email: d0shin03@amc.seoul.kr

Supplementary Figures and legends

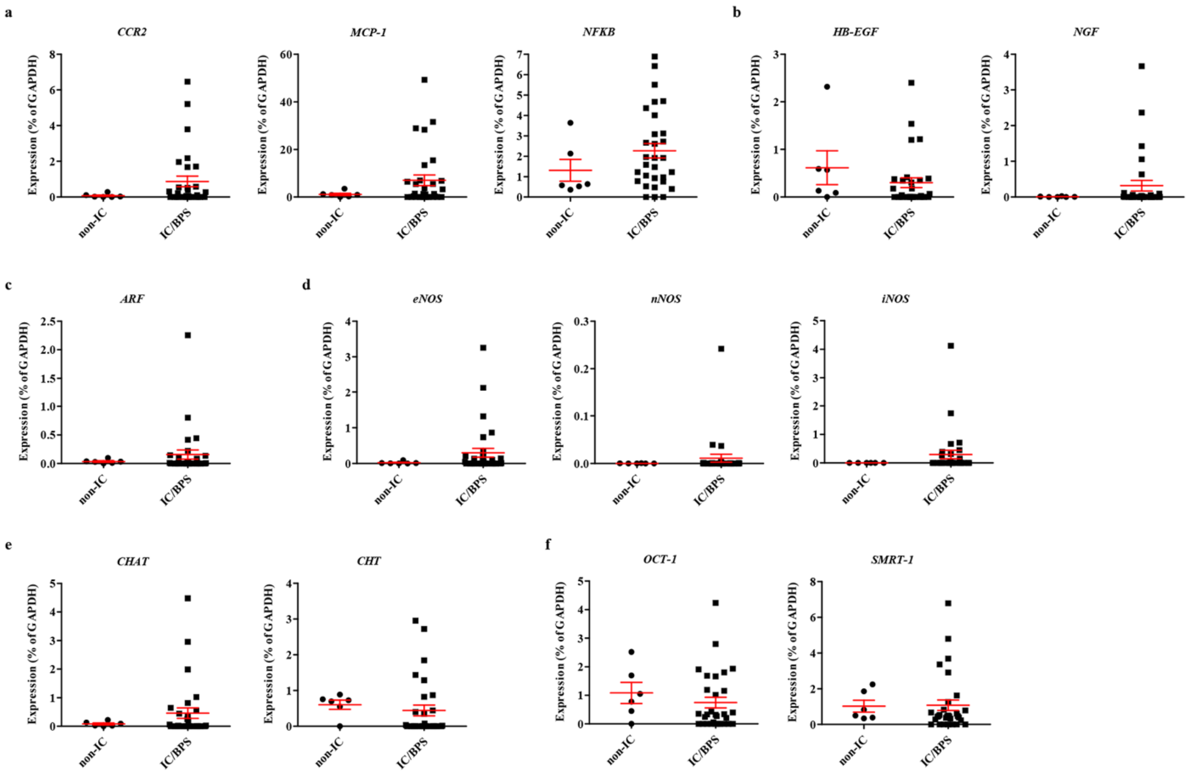

Supplementary Figure 1. Comparison of gene expression associated with IC/BPS pathology between control and IC/BPS patient bladder tissues

(a–f) RQ-PCR data of **Figure 1** regarding inflammation **(a)**, growth factors **(b)**, apoptosis **(c)**, nitric oxide synthase **(d)**, acetylcholine neurotransmitter biosynthesis **(e)**, and transcription regulators **(f)** genes were re-analysed in comparison between control and IC/BPS [non-Hunner-type IC (NHIC) and Hunner-type IC (HIC) patients] groups. Gene expression is presented as a percentage of *GAPDH*. Data are represented as a dot plot of mean ± SEM [n = 6 for non-IC; n = 30 for IC/BPS contain n =11 for NHIC and n = 19 for HIC groups]. non-IC, stress urinary incontinence patients.

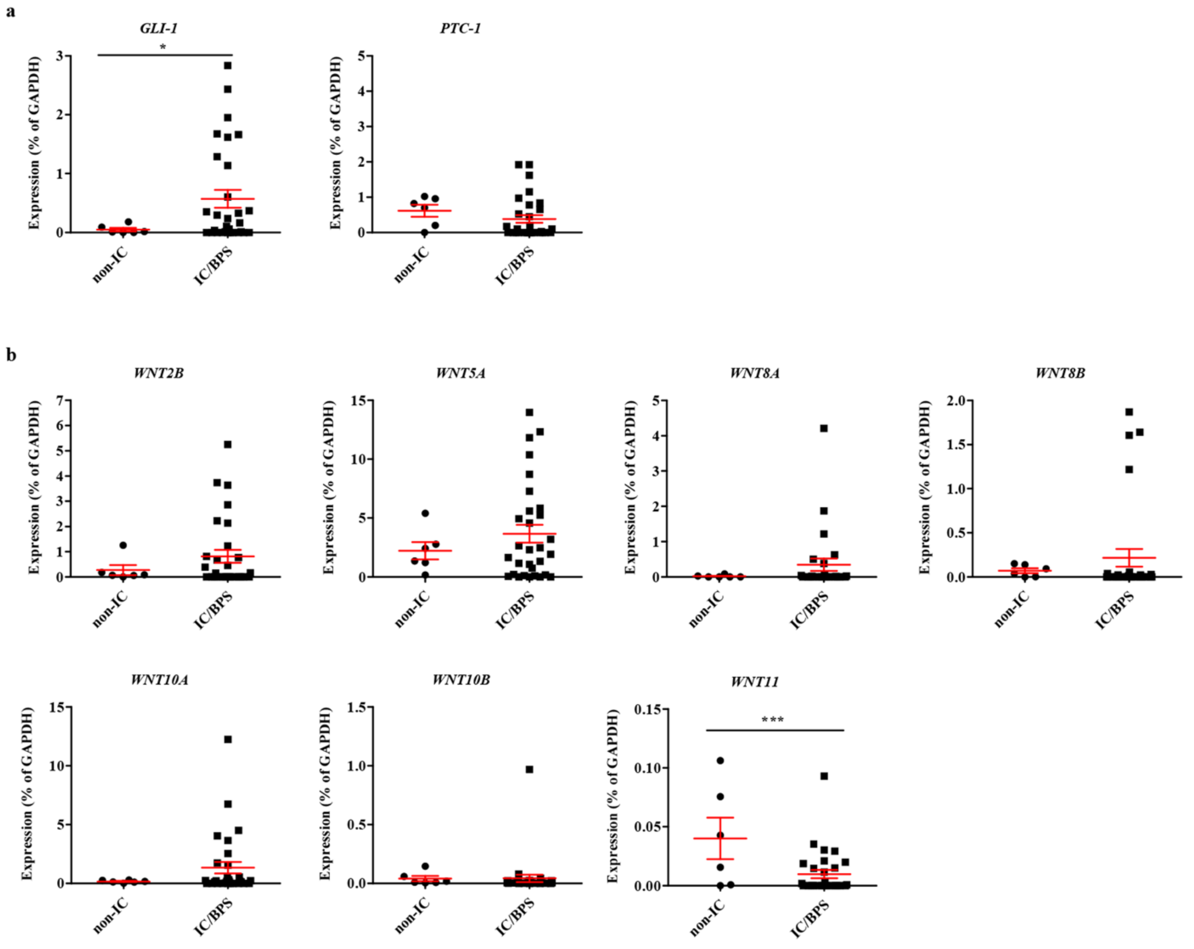

Supplementary Figure 2. Downregulation of *WNT11* in the bladder tissue of IC/BPS patients

(a and b) RQ-PCR data of **Figure 2** regarding SHH **(a)** and WNT **(b)** pathway genes were re-analysed in comparison between control and IC/BPS [non-Hunner-type IC (NHIC) and Hunner-type IC (HIC) patients] groups. Gene expression is presented as a percentage of *GAPDH*. Data are represented as a dot plot of mean ± SEM [n = 6 for non-IC; n = 30 for IC/BPS contain n =11 for NHIC and n = 19 for HIC groups]. non-IC, stress urinary incontinence patients.

a

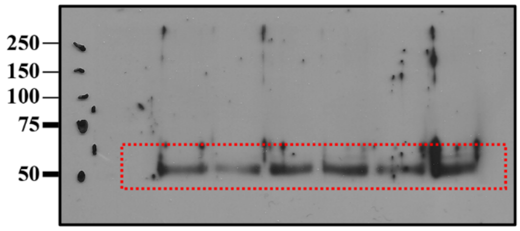

Fig. 8
(p-SMAD2 WB)

b

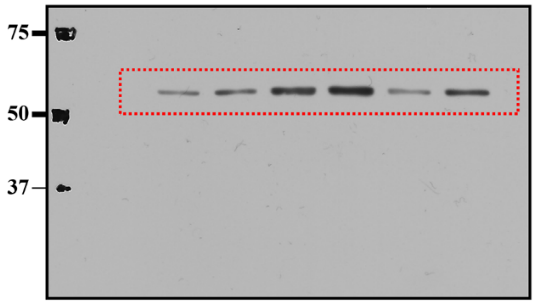

Fig. 8
(VIMENTIN WB)

c

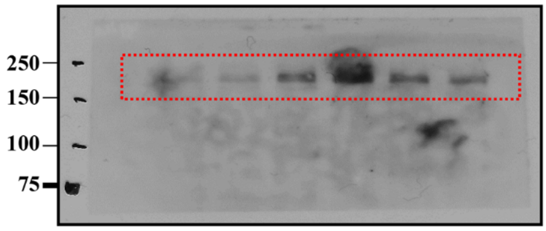

Fig. 8
(FIBRONECTIN WB)

d

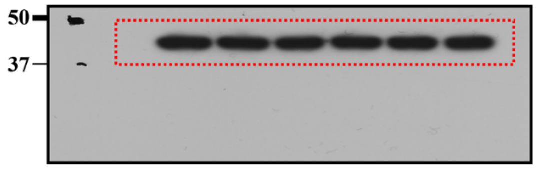

Fig. 8
(β-ACTIN WB)

Supplementary Figure 3. Uncropped western blots

Dotted red line boxes indicate the cropped areas shown in the figures. In all the uncropped western blot images the membranes were simultaneously or sequentially blotted with the indicated antibodies. WB; western blot.
